# Keratinocyte Biomarkers Distinguish Painful Diabetic Peripheral Neuropathy Patients and Correlate With Topical Lidocaine Responsiveness

**DOI:** 10.3389/fpain.2021.790524

**Published:** 2021-12-08

**Authors:** Phillip J. Albrecht, George Houk, Elizabeth Ruggiero, Marilyn Dockum, Margaret Czerwinski, Joseph Betts, James P. Wymer, Charles E. Argoff, Frank L. Rice

**Affiliations:** ^1^Neuroscience and Pain Research Group, Integrated Tissue Dynamics, LLC, Rensselaer, NY, United States; ^2^Division of Health Sciences, University at Albany, Albany, NY, United States; ^3^Department of Neurology, Albany Medical Center, Albany, NY, United States; ^4^Department of Neurology, University of Florida College of Medicine, Gainesville, FL, United States

**Keywords:** neuropathic pain, keratinocytes, IENF, CGRP, sodium channels

## Abstract

This study investigated quantifiable measures of cutaneous innervation and algesic keratinocyte biomarkers to determine correlations with clinical measures of patient pain perception, with the intent to better discriminate between diabetic patients with painful diabetic peripheral neuropathy (PDPN) compared to patients with low-pain diabetic peripheral neuropathy (lpDPN) or healthy control subjects. A secondary objective was to determine if topical treatment with a 5% lidocaine patch resulted in correlative changes among the quantifiable biomarkers and clinical measures of pain perception, indicative of potential PDPN pain relief. This open-label proof-of-principle clinical research study consisted of a pre-treatment skin biopsy, a 4-week topical 5% lidocaine patch treatment regimen for all patients and controls, and a post-treatment skin biopsy. Clinical measures of pain and functional interference were used to monitor patient symptoms and response for correlation with quantitative skin biopsy biomarkers of innervation (PGP9.5 and CGRP), and epidermal keratinocyte biomarkers (Nav1.6, Nav1.7, CGRP). Importantly, comparable significant losses of epidermal neural innervation (intraepidermal nerve fibers; IENF) and dermal innervation were observed among PDPN and lpDPN patients compared with control subjects, indicating that innervation loss alone may not be the driver of pain in diabetic neuropathy. In pre-treatment biopsies, keratinocyte Nav1.6, Nav1.7, and CGRP immunolabeling were all significantly increased among PDPN patients compared with control subjects. Importantly, no keratinocyte biomarkers were significantly increased among the lpDPN group compared with control. In post-treatment biopsies, the keratinocyte Nav1.6, Nav1.7, and CGRP immunolabeling intensities were no longer different between control, lpDPN, or PDPN cohorts, indicating that lidocaine treatment modified the PDPN-related keratinocyte increases. Analysis of the PDPN responder population demonstrated that increased pretreatment keratinocyte biomarker immunolabeling for Nav1.6, Nav1.7, and CGRP correlated with positive outcomes to topical lidocaine treatment. Epidermal keratinocytes modulate the signaling of IENF, and several analgesic and algesic signaling systems have been identified. These results further implicate epidermal signaling mechanisms as modulators of neuropathic pain conditions, highlight a novel potential mode of action for topical treatments, and demonstrate the utility of comprehensive skin biopsy evaluation to identify novel biomarkers in clinical pain studies.

## Introduction

Diabetes continues to be an increasing epidemic as treatment for sequela associated with the disease remain limited (1–4). Current estimates are that over 34 million Americans have diagnosed diabetes, for which approximately one-third (over 10 million) experience sensory dysfunction and painful diabetic peripheral neuropathy (PDPN) (1, 2). A wide range of peripheral neuropathies, including PDPN, share symptoms of tingling and numbness along with severe, intractable chronic pain for which current therapeutic options have little sustained relief, and most have dose limiting and/or debilitating side effects (2, 5–9). Despite extensive research that has identified promising therapeutic interventions, the mechanistic basis of chronic neuropathic pain remains enigmatic (10–13). In the early 2000's, the continued lack of clinical research/development progress in treating chronic pain, including a high failure rate in chronic pain clinical trials, became the rational for creation of the Initiative on Methods, Measurement, and Pain Assessment in Clinical Trials (IMMPACT) group (14). The IMMPACT group specifically recognized that a major issue centered around measures of pain that remain almost entirely based on subjective patient perceptions—which are mired in psychological complications, including placebo effects (15). IMMPACT continues to emphasize the need for more objective, quantifiable biomarker measures with which to validate subjective patient perceptions of pain types, intensities, and therapeutic outcomes. Most recently, IMMPACT has recommended expanding research using skin biopsies as a high-priority means of identifying objective quantifiable biomarkers to validate subjective patient perceptions of pain severity and therapeutic efficacy (16).

Discovered in the late 1980's, a human pan-neuronal enzyme, designated PGP9.5 (PGP), was used to develop an antibody biomarker (17). PGP immunolabeling was the first method to visualize and quantify small-caliber unmyelinated C-fibers that are physiologically and pharmacologically implicated in pain as nociceptors (18, 19). A common quantification, particularly focused on the numerous (and previously unseen) C-fiber terminals in the epidermis, is now referred to as intra-epidermal nerve fibers (IENF) (20). Importantly, in the absence of any prior objective clinical diagnostic to validate chronic pain, PGP immunolabeling of skin biopsy sections provided the first objective visualization of C-fiber pathologies among IENF of patients with a variety of chronic pain afflictions, including PDPN (21, 22). Importantly, the pathology involves a paradoxical reduction in the density of the presumed nociceptor IENF, clinically diagnosed as small fiber neuropathy (SFN) (7, 8, 22–25).

A predominant hypothesis for this paradox is the “irritable nociceptor” which posits that the remaining C-fibers have undergone a structural and/or neurochemical pathological change that renders them hypersensitive to noxious or even non-noxious stimuli (11, 26–28). Consistent with this hypothesis is electrophysiological evidence of hyperexcitable and spontaneous activity among C-fibers and DRG neurons of chronic pain patients (29–33). The pathological mechanisms involved in nociceptor hypersensitivity continue to be elusive, although alterations in the expression of voltage-gated sodium channels (Nav) and a role of calcitonin gene-related peptide (CGRP) are known to be involved (29, 34–46).

PGP immunodetection of IENF has become the accepted diagnostic of SFN in chronic pain, however PGP has no known direct effect on neuronal signaling as a mechanistic biomarker and does not consistently validate subjective patient perceptions of chronic pain intensity (7, 24, 25, 30, 47–53). For example, some patients with chronic painful neuropathy do not show SFN, whereas SFN has been detected among many patients with non-painful neuropathy (6, 22, 54, 55). Consistent with the IMMPACT recommendations for expanded research on skin biopsies to identify reliable objective chronic pain biomarkers, we developed a comprehensive evaluation platform, referred to as chemomorphometric analysis (CMA), that extensively uses PGP immunolabeling among combinations of immunolabels for numerous functionally implicated neuronal signaling molecules. By varying the combinations among alternating serial sections, CMA has enabled comprehensive biomarker profiling of normal, experimental, and pathological specimens from human, monkey, and a variety of rodent and non-primate mammalian species (7, 36, 56–60).

Our first objective was to use CMA on skin biopsies in an open-label proof-of-principle clinical trial to determine if mechanistically-relevant pain biomarkers had quantitative correlation to subjective pain perception among diabetic patients ranging from high to low level neuropathy pain as compared to normal subjects. Based on the evidence for Nav involvement in chronic pain, our second objective used topical 5% lidocaine patch applications to test whether any of the patients received clinical benefit that correlated with the quantifiable biomarkers measured with CMA from pre-treatment and post-treatment skin biopsies. The skin biopsy CMA focused on quantifying biomarkers among two components: the C-fiber innervation and the epidermal keratinocytes. Expanding on prior quantification of PGP for IENF in diabetic patients, our analysis distinguished between two major functionally different types of C-fiber – peptidergic and non-peptidergic – that might be differentially affected as a potential marker of chronic pain (6, 25, 47, 48). Both C-fiber types label for PGP and are implicated as nociceptors that drive pain perception (61, 62). Peptidergic C-fibers also immunolabel for CGRP, a potent vasodilator released from sensory endings that mediates neurogenic inflammation and plays critical roles in pain, including migraine (7, 40, 63–65). These small caliber C-fibers may be differentially vulnerable to pathological diabetic conditions such as prolonged hyperglycemia, insulin resistance and insufficiency, the generation of reactive oxygen species (ROS), advanced glycation end-products acting via their receptor (RAGE), polyol pathway utilization dysfunction, and protein kinase C activation (53, 66–70). Therefore, in addition to CGRP measures, the innervation quantification included IENF and density in the upper dermis to determine if variable effects on innervation could be detected among the capacity of C-fiber regeneration as a biomarker for chronic pain.

Recent optogenetic evidence has now confirmed prior immunolabeling studies implicating epidermal keratinocytes in modulating the sensitivity of C-fibers through an expression and release of a mixed variety of neurotransmitters involved in algesic, analgesic, and pruritic mechanisms (36, 44, 62, 71–82). In particular, disruptions of these keratinocyte signaling properties, especially increases in Nav1.6, Nav1.7, and CGRP expression have been observed in several painful human conditions, as well as in a diabetic monkey population, concomitant with significant disarray of cutaneous innervation (7, 36, 44, 58, 62, 72). Therefore, relative intensities of immunofluorescence labeling for Nav1.6, Nav1.7, and CGRP among the epidermal keratinocytes was assessed as other potential objective biomarkers of subjective patient perceptions of pain intensity (7, 62, 81).

## Methods

### Clinical Research Study

All clinical research portions of this study were conducted under IRB approval from Albany Medical College (2008–2324) and all clinical procedures were carried out at Albany Medical Center, Albany, NY, USA. Each subject was informed of the entire study process and consented to all testing parameters, procedures, and use of biopsy material for research purposes. The open-label clinical research study was registered with ClinicalTrials.gov (identifier number: NCT01086150) as a means to increase enrollment.

### Subjects

The study was designed to enroll 40 patients (30 with PDPN, 10 with lpDPN) and 10 age- and gender-matched control subjects. All enrolled patients met the Toronto criteria for confirmed clinical diabetic sensorimotor polyneuropathy (83). The final cohorts for analysis consisted of: 1) 21 adults aged 34–67 years with previously diagnosed PDPN, and a pre-treatment (PRE) VAS score defined by clinically significant pain (~>3/10), 2) 12 adults aged 47–72 years with previously diagnosed diabetic peripheral neuropathy without evidence of clinically significant pain (lpDPN), and 3) 11 healthy subjects aged 30–62 years with no history of painful neuropathy or analgesic medication use (Control; see Table 1). All diabetic patients had stable glycemic control, and none had any co-morbidities associated with peripheral neuropathies, unstable medical conditions, or medications that would be expected to affect sodium channel function. After eligibility was determined, all study subjects (controls and patients) underwent a general neurological evaluation, and quantitative sensory testing (QST). The patient groups (lpDPN, PDPN) also gave an initial site visit visual analog scale (VAS) measure of pain intensity, and completed the Brief Pain Inventory (BPI) for functional pain and daily life interference, the Neuropathic Pain Scale (10 score), the Neuropathy Impairment Score in the Lower Limbs (NIS-LL), and the Neurological Symptom Scale (NSS).

**Table 1:** Total cohort data summary.

**Ave (min, max)**	**Control**	**lpDPN**	**PDPN**	**Between-group comparisons**
**Demographic**	***n* = 11**	***n* = 12**	***n* = 21**	**Correlation**
Gender	6F, 5M	4F, 8M	9F, 12M	No difference
Age (yrs)	46 (30, 62)	58 (47, 72)	53 (34, 67)	*Significant*: Control < lpDPN
Age at diabetes onset (yrs)	n/a	46 (40, 63)	42 (19, 60)	No difference
Diabetes duration (yrs)	n/a	12 (1, 26)	12 (1, 41)	No difference (Wilcoxon-Mann-Whitney)
Neuropathy duration (yrs)	n/a	4 (1, 8)	12 (2, 32)	No difference (Wilcoxon-Mann-Whitney)
BMI (in/lbs)	27.5 (19.7, 34.1)	34.9 (23.9, 47.9)	33.2 (22.4, 58.3)	No difference (Wilcoxon-Mann-Whitney)
HbA1c	5.6 (5.1, 5.8)	7.2 (5.8, 10.6)	8.0 (6.6, 9.6)	*Significant*: Control < lpDPN, PDPN
Systol bp (mm Hg)	113 (100, 126)	124 (100, 136)	124 (100, 148)	*Significant*: Control < PDPN
Diastol bp (mm Hg)	75 (66, 86)	76 (68, 100)	74 (60, 92)	no difference
**Questionnaire/Diary**				**Student's** ***T*****-test or Wilcoxon-Mann-Whitney**
BPI - FPSI (0-10) PRE	n/a	0.6 (0.0, 2.5)	5.7 (0.0, 8.8)	*p < * 0.01 (Wilcoxon-Mann-Whitney)
BPI - FPSI (0-10) POST	n/a	0.5 (0.0, 1.8)	4.3 (0.0, 9.0)	*p < * 0.01 (Wilcoxon-Mann-Whitney)
BPI - DLII (0-10) PRE	n/a	0.5 (0.0, 3.7)	4.6 (0.0, 9.3)	*p < * 0.01 (Wilcoxon-Mann-Whitney)
BPI - DLII (0-10) POST	n/a	0.3 (0.0, 2.3)	3.9 (0.0, 9.3)	*p < * 0.01 (Wilcoxon-Mann-Whitney)
NPS[10] (0-100) PRE	n/a	8.8 (0.0, 26.0)	56.0 (22.0, 95.0)	*p < * 0.01 (Wilcoxon-Mann-Whitney)
NPS[10] (0-100) POST	n/a	7.5 (0.0, 29.0)	43.9 (0.0, 72.0)	*p < * 0.01 (Wilcoxon-Mann-Whitney)
NIS-LL (0-88) PRE	n/a	13.0 (0.0, 38.0)	11.9 (6.0, 32.0)	n.s.
NIS-LL (0-88) POST	n/a	11.1 (0.0, 42.0)	10.8 (2.0, 32.0)	n.s.
NSS (0-31) PRE	n/a	5.3 (1.0, 16.0)	10.2 (1.0, 22.0)	*p < * 0.01 (Student's *T*-test)
NSS (0-31) POST	n/a	6.1 (1.0, 17.0)	10.0 (2.0, 17.0)	*p < * 0.05 (Student's *T*-test)
Sleep Interference (0-10) PRE	n/a	1.0 (0.0, 3.0)	5.8 (1.0, 10.0)	*p < * 0.01 (Student's *T*-test)
Sleep Interference (0-10) POST	n/a	0.2 (0.0, 1.0)	3.7 (0.0, 9.0)	*p < * 0.01 (Wilcoxon-Mann-Whitney)
Composite Clinical Score PRE	n/a	5.3 (1.0, 13.0)	16.0 (6.0, 25.0)	*p < * 0.01 (Wilcoxon-Mann-Whitney)
Composite Clinical Score POST	n/a	4.6 (1.0, 13.0)	13.0 (1.0, 23.0)	*p < * 0.01 (Wilcoxon-Mann-Whitney)
Composite VAS (0-10) PRE	n/a	1.2 (0.0, 3.1)	6.5 (4.5, 9.0)	*p < * 0.01 (Wilcoxon-Mann-Whitney)
Composite VAS (0-10) POST	n/a	0.5 (0.0, 1.5)	4.5 (0.0, 8.9)	*p < * 0.01 (Wilcoxon-Mann-Whitney)
**Quantitative Sensory Testing (QST)**				**ANOVA or Kruskal-Wallis**
Cold sense (°C) PRE	25.7 (17.2, 29.7)	23.0 (6.0, 29.0)	19.4 (2.2, 27.7)	*p < * 0.05 - Control vs. PDPN
Cold sense (°C) POST	24.1 (16.4, 28.7)	24.4 (21.0, 36.7)	20.6 (6.5, 27.2)	n.s.
Warm sense (°C) PRE	38.1 (34.3, 41.5)	43.2 (36.2, 49.5)	44.8 (37.9, 50.9)	*p < * 0.01 - Control vs. lpDPN, PDPN
Warm sense (°C) POST	39.2 (35.9, 43.6)	43.1 (36.7, 48.2)	44.6 (36.2, 50.1)	*p < * 0.05 - Control vs. lpDPN; *p < * 0.01 - Control vs. PDPN
Heat pain (°C) PRE	47.6 (40.7, 50.9)	49.8 (46.3, 52.0)	49.2 (43.9, 51.8)	*p < * 0.05 - Control vs. lpDPN
Heat pain (°C) POST	47.1 (44.1, 49.4)	49.6 (45.4, 52.5)	50.3 (39.2, 54.4)	*p < * 0.05 - Control vs. lpDPN; *p < * 0.01 - Control vs. PDPN
**ChemoMorphometric (CMA)**				**ANOVA or Kruskal-Wallis**
PGP EPI entry (per mm) PRE	1.8 (0.2, 4.3)	0.5 (0.0, 2.6)	0.6 (0.0, 3.7)	*p < * 0.05 - Control vs. lpDPN; *p < * 0.01 - Control vs. PDPN
PGP EPI entry (per mm) POST	2.1 (0.4, 4.6)	0.7 (0.0, 3.3)	0.5 (0.0, 2.9)	*p < * 0.05 - Control vs. lpDPN; *p < * 0.01 - Control vs. PDPN
PGP EPI endings (per mm) PRE	3.6 (0.4, 6.4)	1.1 (0.0, 5.2)	0.9 (0.0, 6.0)	*p < * 0.05 - Control vs. lpDPN; *p < * 0.01 - Control vs. PDPN
PGP EPI endings (per mm) POST	4.1 (0.4, 8.7)	1.7 (0.0, 7.7)	0.8 (0.0, 7.1)	*p < * 0.05 - Control vs. lpDPN; *p < * 0.01 - Control vs. PDPN
PGP Subepidermal (per mm) PRE	4.1 (2.2, 6.1)	2.0 (0.5, 5.0)	1.6 (0.3, 4.5)	*p < * 0.01 - Control vs. lpDPN, PDPN
PGP Subepidermal (per mm) POST	3.9 (1.8, 6.7)	1.5 (0.3, 5.3)	1.5 (0.0, 4.7)	*p < * 0.01 - Control vs. lpDPN, PDPN
PGP Upper Dermal (per mm) PRE	13.3 (8.7, 20.1)	11.6 (3.3, 16.5)	8.6 (3.6, 16.7)	*p < * 0.01 - Control vs. PDPN
PGP Upper Dermal (per mm) POST	13.5 (9.4, 17.2)	10.9 (4.0, 15.5)	8.8 (2.9, 23.0)	*p < * 0.05 - Control vs. PDPN
CGRP Subepidermal (per mm) PRE	0.63 (0.12, 1.57)	0.35 (0.00, 0.65)	0.36 (0.00, 2.06)	*p < * 0.05 - Control vs. PDPN
CGRP Subepidermal (per mm) POST	0.86 (2.00, 0.12)	0.28 (0.00, 1.21)	0.29 (0.00, 1.62)	*p < * 0.05 - Control vs. lpDPN, PDPN
CGRP Upper Dermal (per mm) PRE	5.12 (1.85, 8.69)	4.50 (1.06, 9.00)	3.74 (0.79, 8.43)	n.s.
CGRP Upper Dermal (per mm) POST	6.90 (1.52, 12.40)	4.35 (1.90, 7.83)	2.94 (0.60, 8.41)	*p < * 0.05 - Control vs. PDPN
Keratinocyte Nav1.6 (PI) PRE	81.2 (63.54, 127.38)	87.9 (49.30, 126.44)	101.4 (64.69, 135.50)	*p < * 0.05 - Control vs. PDPN
Keratinocyte Nav1.6 (PI) POST	91.0 (49.19, 132.37)	90.8 (60.38, 148.07)	90.7 (60.85, 134.63)	n.s.
Keratinocyte Nav1.7 (PI) PRE	47.5 (30.84, 73.33)	54.3 (40.66, 66.09)	59.6 (39.41, 87.53)	*p < * 0.05 - Control vs. PDPN
Keratinocyte Nav1.7 (PI) POST	52.4 (39.82, 85.83)	54.7 (34.43, 77.57)	51.5 (30.0, 81.35)	n.s.
Keratinocyte CGRP (PI) PRE	27.7 (22.37, 32.83)	29.5 (19.51, 39.83)	38.5 (27.49, 68.14)	*p < * 0.01 - Control vs. PDPN
Keratinocyte CGRP (PI) POST	31.5 (21.86, 49.53)	26.2 (20.52, 36.58)	32.8 (19.03, 56.10)	n.s.

*Complete group data averages and ranges for all endpoint values and between group statistical analysis results*.

Following the patient clinical assessments, all study subjects (controls and patients) had a 3 mm glabrous skin punch biopsy collected from the lateral margin of one foot, about half-way between the heel and base of the fifth toe, where QST testing was performed. The lateral foot site was chosen because it is a more prevalent and intense pain location reported by diabetic patients as compared to the more proximal location 5 cm above the lateral malleolus that has become a standard biopsy site used for SFN diagnosis in most human chronic pain afflictions, including PDPN. Moreover, the lateral foot has a greater variety and concentration of peripheral innervation, including large-caliber myelinated non-painful tactile varieties of innervation that are also impacted by diabetes, making this location optimal for QST testing. Finally, the lateral foot has a higher concentration of microvascular arbors and neurovascular innervation. While not included in this report, the myelinated innervation and neurovascular components within the same biopsies will be the subject of future studies. All study subjects were subsequently monitored for 7 days following biopsy collection and patient groups kept daily diary NRS records of pain and sleep disturbance prior to beginning topical treatment (pre-treatment period; see Study Design).

After the 1-week pre-treatment period, all study subjects returned to the clinic, healthy control subjects were screened for any difficulties from biopsy collection or changes in pain status, of which none were reported. Of the initial 40 diabetic subjects enrolled and biopsied at baseline, 33 continued onto the study drug phase. One patient was removed for increased glucose levels, and 6 withdrew for various undisclosed reasons. None of the withdrawals were associated with negative consequences of pre-treatment biopsy collection, there were no reports of negative consequences following the pre- or post-treatment biopsies, and all patients were not in a stage of disease with any tissue healing issues or loss of tissue integrity. The patient groups completed a VAS measure, and after which the patients and healthy subjects initiated applications of a 5% lidocaine patch (Lidoderm®, Endo Pharmaceuticals) directly to the area of foot pain for those with PDPN (including the biopsy site location) or over the lateral dorsum (including the biopsy site) of the foot for those with lpDPN and controls, using a maximum of 4 patches per day (2 every 12 h) over a 4-week topical treatment protocol. Following completion of the 4-week treatment period, repeat neurological examination, QST, VAS, BPI, NPS, NIS-LL, and NSS were performed/administered to the patient groups, and all study subjects had a post-treatment skin biopsy collected at a site 1 cm proximal to the initial biopsy site (see Study Diagram). Patients that showed a ≥30% reduction in VAS scores (responders, see below) were allowed to continue using the patch and were monitored by phone for negative side-effects for up to 1 year, during which no negative side-effects were reported.

**Study Diagram:**
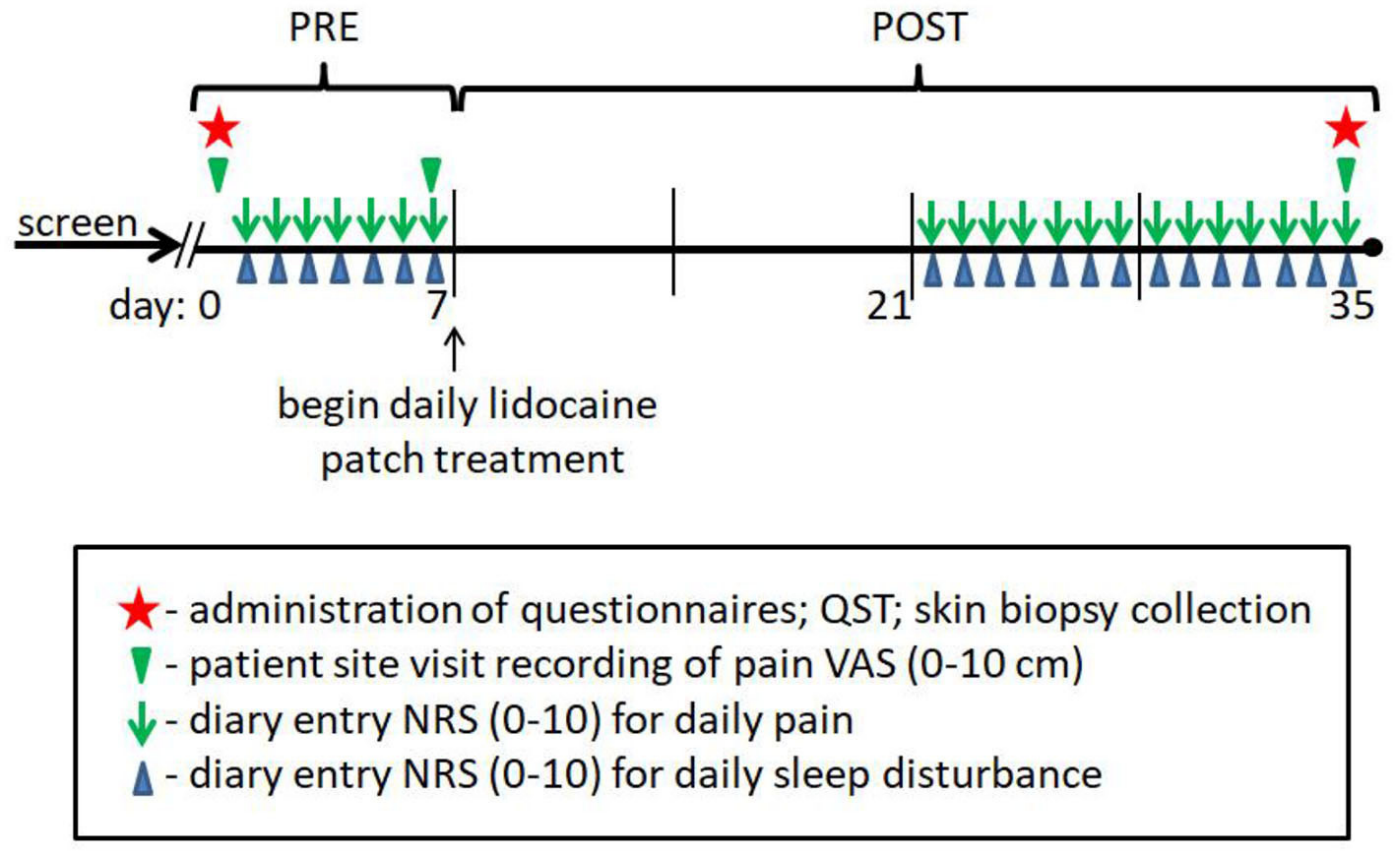
Visual outline of the study design and timeframe for subject participation.

### Clinical Measures

Standard demographic (age, gender, ethnicity) and clinical data (BMI, blood pressure, HbA1c), and quantitative sensory testing (QST) were collected from all study subjects, and subjective clinical measures (VAS, BPI, NPS10, NIS-LL, NSS, as defined below) were obtained from the PDPN and lpDPN patients to assess pain and functional symptoms at pre-treatment and after the 4-week topical treatment protocol. Daily pain and sleep disturbance diaries (NRS; 0-10) were maintained during the 1-week pre-treatment period and during the 4-week patch treatment period (see Study Diagram).

#### QST

Quantitative Sensory Testing was performed at the lateral foot using standardized methods with values for cold and warm detection and heat pain thresholds used for determination of small fiber functionality (84).

#### VAS

The Visual Analog Score (VAS) is a simple measure of subjective pain rated on scale of 0–100 (or 0–10) with terms of no pain (0) through worst pain imaginable (100 or 10), presented here as a simple 100 mm line for patients to mark at the distance that corresponds to the level of their perceived pain.

#### NRS

Numerical Rating Score (0-10; 11-point scale) were used for the daily diary pain measures, as no pain (0) through worst pain (10). The sleep disturbance scores were marked in daily morning (AM hours) diary logs with terms of no disturbance (0) through complete disturbance (10). Sleep disturbance scores were averaged over the pre-treatment period and the post-treatment period to get a single estimate (composite sleep score) of the overall sleep disturbance the participants were experiencing prior to, and after, treatment.

#### BPI

The Brief Pain Inventory (BPI) records the subjective pain experience during the previous 24 hr (functional pain severity index), as well as the level to which pain interfered with normal routines (daily life interference index). Pain severity and pain interference are rated from 0 (no pain/interference) to 10 (incapacitating pain/interference). The BPI is a self-administered single page questionnaire addressing how the patient perceives the pain and the degree that the pain caused interference with normal daily activity, mood, work, interpersonal relations, sleep, and overall quality of life. Reports demonstrate strong internal consistency and reliability for the BPI in patients with chronic pain and diabetic neuropathy (85, 86).

#### NPS10

The Neuropathic Pain Scale measures the following 10 aspects of pain, each on a scale of 0–10 (0 = no pain, 10 = the worst pain imaginable): intensity, unpleasantness, hot, cold, sharp, dull, itchy, skin sensation (sensitivity), surface pain, and deep pain. The NPS Composite Score (NPS 10) is the sum of all 10 NPS descriptors (total scale of 0–100). Although the NPS can be used to further render multidimensional aspects of neuropathic pain into several different composite scores (i.e., NPS8 or NPS4), we used the full composite NPS10 scores to evaluate responsiveness.

#### NIS-LL

The Neuropathy Impairment Score is a composite clinical scoring system used to measure the functional impairment of peripheral neuropathy, and the NIS-LL is a lower limbs specific subset of the NIS used to evaluate the location which most often presents the earliest symptom onset in PDN (87). The NIS-LL scores clinical abnormalities of sensation, muscle power, and tendon reflexes following neurologic examination (from 0 = normal to 88=total impairment). The three components of the NIS-LL measure different functionality of the peripheral nervous system: *Sensation* (pressure, pinprick, vibration, joint position). The components of the sensory examination, except for joint position, are assessed on the dorsal surface at the base of the right and left great toenails. Joint position is assessed by moving the terminal phalanx of the right and left great toes. Sensory assessment is scored as 0 (normal), 1 (decreased), or 2 (absent). Four assessments are performed bilaterally creating a maximum possible score of 16. *Reflexes*. Reflex assessment is scored as 0 (normal), 1 (decreased), or 2 (absent), with adjustments made for the patient age (i.e., patient older than 65 years with decreased response is still assessed as 0, or normal). Two assessments are performed bilaterally creating a maximum possible score of 8. *Muscle weakness*. Muscle weakness is scored as 0 (normal), 1 (25% weak), 2 (50% weak), 3 (75% weak), 3.25 (move against gravity), 3.5 (movement, gravity eliminated), 3.75 (muscle flicker, no movement), or 4 (paralysis). Eight assessments are performed bilaterally with a maximum possible score of 64.

#### NSS

Neuropathy Symptom Score (NSS) is a clinical scoring scale that measures the positive and negative symptoms associated with neuropathy. The questionnaire asks the patient 31 questions based on 3 general symptom groups, 1) muscle weakness in various muscle groups or while performing daily tasks, 2) sensory disturbances such as difficulty with identifying objects in the mouth or hand or burning/numbing sensations at any location, and 3) autonomic symptoms such as fainting or abnormal appetite, sweating or eye sensitivity. The score is the sum of all “yes” responses (0–31), with a higher number indicating an increased degree of neuropathy.

### Tissue Morphology Measures

#### Biopsy Processing

All biopsies were processed, immunolabeled, and evaluated following standard INTiDYN ChemoMorphometric Analysis (ITD-CMA™) protocols using routine published methodologies (57–59, 88, 89). Upon arrival in the laboratory, each biopsy was given a data ID number that was separate from any clinical identification, such that no HIPPA-sensitive personal identifiers were associated with the data set. All biopsy processing, immunolabeling, data collection, and analyses were performed under fully-blinded conditions. Immediately following collection, skin biopsies were immersion fixed for 4 h at 4°C in freshly prepared 4% paraformaldehyde in 0.1 M phosphate buffered saline (PBS, pH7.4), then rinsed and stored in fresh PBS under refrigeration. Biopsies were subsequently infiltrated with 30% sucrose in PBS overnight, mounted in Optimal Cutting Temperature (OCT) gel, frozen, and sectioned by cryostat at 14 μm thickness. Sections were thaw mounted in serial order with alternating sections rotated across 20 slides such that each slide contains up to 20 sections equally spaced through the entire biopsy. Slides were then air-dried overnight at room temperature. Subsequently, slides were rehydrated and preincubated with PBS containing 1% bovine serum albumin and 0.3% Triton-100X (PBS-T) for 30 min at room temperature. Primary antibodies were diluted in PBS-T and separate slides from each biopsy were immunolabeled for the pan-neuronal enzyme PGP (rabbit polyclonal, 1:800, UltraClone Ltd.) to visualize and quantify total cutaneous innervation, and for Nav1.6 (rabbit polyclonal, 1:100; Alomone Labs), Nav1.7 (rabbit polyclonal, 1:100; Alomone Labs), and CGRP (sheep polyclonal, 1:500, Abcam), to determine epidermal keratinocyte immunolabeling. Slides were incubated under primary antibodies for one night with refrigeration. Immunolabeling for PGP and CGRP innervation, and Nav1.6, Nav1.7, and CGRP expression and immunolabeling among keratinocytes has been well-validated and previously published by our group (7, 36, 44, 56, 57, 59, 60).

Following primary antibody incubations, slides were rinsed in excess PBS and then incubated with appropriate species secondary antibodies conjugated with either Cy3 (red fluorescence) or Alexa488 (green fluorescence) diluted 1:500 in PBS-T at room temperature for 2 hrs. The DNA binding protein DAPI (4',6-Diamidino-2-Phenylindole) was also included in the secondary antibody mix to stain cell nuclei (blue fluorescence). After secondary antibody labeling, slides were rinsed in excess PBS and mounted under coverslips with 90% glycerin in PBS with 0.05% sodium azide, and stored at −20°C until analysis.

#### Epifluorescence Microscopy

Epifluorescent 20x microscopy image montages of PGP immunolabeling from each biopsy were captured and used to quantify the cutaneous innervation with Neurolucida software (v9, MBF Biosciences, Essex, VT) mapping routines, including epidermal, subepidermal, and upper dermal compartments. For epidermal keratinocyte assessments, Nav1.6, Nav1.7, and CGRP epifluorescence microscopy images were obtained with a Hamamatsu ER DVC high-speed camera and linear focus encoder which was also operated with the Neurolucida software package. At least 5 non-edge and non-overlapping fields from 3 separate sections for each biomarker assessment were captured at identical camera settings and average pixel intensity (PI) values of *n* = 15 measures per biopsy analyzed using the freely available ImageJ software (v1.51).

### Data Analysis

**Figure 1:**
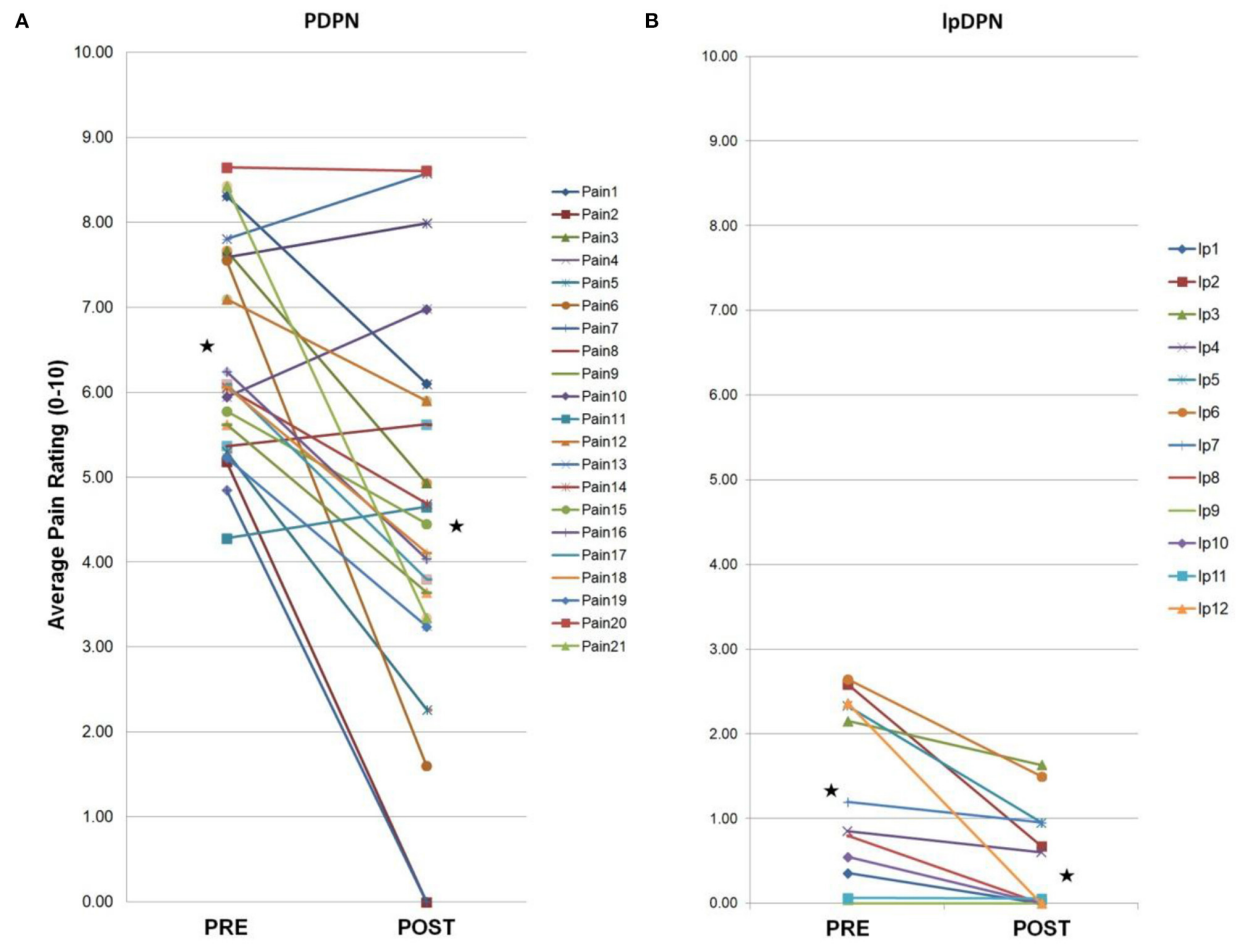
Individual composite pain scores. A 4-week topical 5% lidocaine treatment resulted in a significant reduction in average pain scores for the PDPN and lpDPN cohorts. Pre-treatment (PRE) and post-treatment (POST) composite pain scores are shown for **(A)** the PDPN (Pain 1–Pain 21) and **(B)** the lpDPN (lp1-lp12) patient cohorts. Black stars represent the group average values with significant differences (*p* ≤ 0.05 by Wilcoxon-Mann-Whitney).

**Figure 2:**
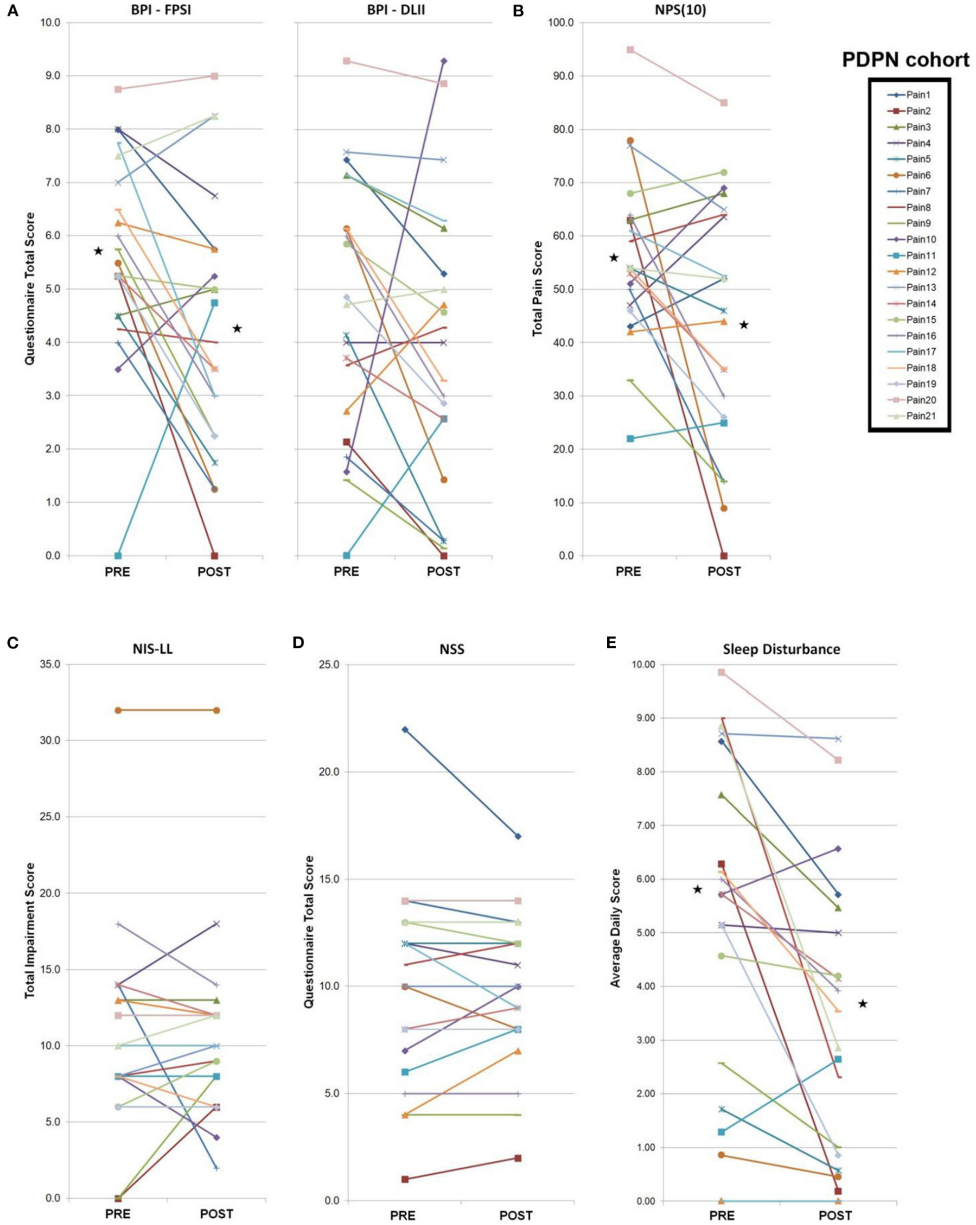
PDPN cohort individual clinical measures. Total PDPN cohort (*n* = 21) individual clinical symptoms responses (questionnaires, measures, composite sleep disturbance). Black stars represent the group average values with significant differences (*p* < 0.05 by paired Student's *t*-test). **(A)** Following a 4-week topical 5% lidocaine treatment, significant improvements were observed on the BPI-FPSI, but not the BPI-DLII. **(B)** Significant improvements were observed on the NPS (10) score. **(C)** No significant group changes were observed for the NIS-LL score, or **(D)** the NSS score. **(E)** Significant improvements were observed in group composite sleep disturbance scores.

Group averages for demographic data, clinical questionnaire and diary response data, QST responses, and the CMA data for innervation and keratinocyte pixel intensity (PI) were analyzed across the three cohort groups (Control, lpDPN, PDPN) using Microsoft Excel (v2010) with Analyze-it (v5.9; Leeds, UK) add-in functions. Collected data sets that passed the Anderson-Darling normality test were analyzed for between-group comparisons by parametric methods (Student's *T*-test, or ANOVA with Tukey-Kramer *post-hoc* testing) and those without normality were compared by non-parametric methods (Wilcoxon-Mann-Whitney, or Kruskal-Wallis with Steel-Dwass-Critchlow-Fligner *post-hoc* testing), as shown (Table 1). For within-group comparisons, paired *T*-tests or Wilcoxon-Mann-Whitney tests were performed to compare PRE/POST changes for composite VAS scores and each subjective clinical response measure (see Figures 1, 2). Baseline data from dropout patients was not included in any analysis. Correlation analyses (SAS, v9.4) were also run using the demographic data, measured biomarkers from CMA, and clinical responses intent to define relationships between the variables and to determine if any subjective patient responses were correlated with quantifiable biomarkers from pre-treatment biopsies. A liberal criterion was used for flagging possible correlations; if a single correlation had a *p* < 0.05, it was retained and the analysis re-run. If there were no correlations reaching the significance level, they were dropped from subsequent runs. To evaluate the extent to which skin biopsy markers were able to differentiate the three groups (Control, lpDPN, PDPN) prior to implementation of treatment, a MANOVA was used (90). The pre-treatment biopsy variables that were evaluated included keratinocyte immunolabeling intensities for Nav1.6, Nav1.7, CGRP, and the PGP innervation marker counts (EPI entry, EPI endings, Subepidermal, and Upper Dermal) across groups. To follow up on significance, a Bonferroni adjustment was made when evaluating differences between groups on all outcome variables.

To evaluate the post-treatment differences between groups on the biopsy markers, a MANCOVA was performed using all pre-treatment variables as covariates for statistical control in evaluating post-treatment effects on the biopsy markers (90). Composite pain scores were compiled for each patient making use of the site visit VAS scores and the diary entry NRS scores (see Study Diagram). The pre-treatment composite pain score (Figure 1) represents the average of 9 measures (2 site visit VAS and 7 NRS daily diary entries) and the post-treatment composite pain score comprised the average of 15 values (14 NRS scores from daily diary during the final 2 weeks of treatment and 1 final site visit VAS). To evaluate the extent to which pre-treatment variables could predict effective response to the treatment, the patient group was partitioned into a set of responders and non-responders. Responders were identified as individuals with a 30% or more reduction in subjective pain rating from the pre-VAS scores to the post-VAS score. Pre-VAS scores were averaged to get a single estimate of the overall pain the participants were experiencing prior to treatment. The post-VAS score was used as the overall pain the participants were experiencing after the treatment regime. The following formula was used to identify an individual as a responder:


(1) $$\left(\frac{\text{preVAS-postVAS}}{\text{preVAS}}\right)\geq \text{0}.\text{30}$$

and responders were positively coded, otherwise the individual was identified as a non-responder. To evaluate pre-treatment variables for their predictive utility in identifying treatment responders, a logistic regression model was used (91).

## Results

No adverse reactions were observed as a result of the biopsy procedures or during the 4-week topical 5% lidocaine treatment protocol and no subjects withdrew due to negative side-effects associated with treatment.

### Clinical Analysis

Table 1 provides descriptive statistics of the sample (*N* = 44). There were 11 control, 12 lpDPN, and 21 PDPN group subjects. Correlative results indicated that gender across the groups was not significantly different, χ^2^[2] = 1.05, *p* = 0.59. One subject identified as a person of color, and all others identified as Caucasian. Ages between groups were statistically significant, *F*_(2, 41)_ = 5.22, *p* < 0.01. Follow up analysis adjusting for multiple tests indicated that the control group (*M* = 46.18) was significantly younger than the lpDPN group (*M* = 58.58). No differences were found between the age of diabetes onset, diabetes duration, or neuropathy duration between the lpDPN and PDPN patient groups. No significant differences were found between groups on BMI, *F*_(2, 41)_ = 2.52, *p* = 0.09. Significant differences were seen between groups on HbA1c, *F*_(2, 40)_ = 23.66, *p* < 0.01. Follow up analyses, correcting for multiple tests, indicated that the control group was significantly lower than both the lpDPN and PDPN groups, but no significant difference was found between the lpDPN and PDPN groups. Significant differences were noted on systolic BP, *F*_(2, 41)_ = 3.95, *p* < 0.05, and follow up analyses, correcting for multiple tests, found the PDPN group was significantly higher than the control group. No significant differences were found between groups on the diastolic BP measure. Additional correlational analysis demonstrated that neuropathy duration, previous use of anti-cancer, anti-coagulant, anti-depressant, NSAID, or sleeping aid drugs, or previous/current comorbid allergy/immune, asthma, migraine, psychiatric, or thyroid disorders were not correlated with any pre-treatment biopsy measures. In contrast, weight, BMI, systolic BP, HbA1c, previous use of anti-hypertensives, gabapentin, or narcotics, or previous/current comorbid gastrointestinal, hypertension, or musculoskeletal disorders had at least one significant correlation (*p* < 0.05). These additional correlative results will be followed-up in future studies.

Composite pain scores (Figure 1) demonstrate all of the pre-diagnosed PDPN patients had an average pre-treatment pain rating score ≥3/10 (group average = 6.5), indicative of clinically-meaningful pain, and all of the pre-diagnosed lpDPN patients had an average pre-treatment pain rating score <3/10 (group average = 1.2). Additionally, each of the pre-treatment clinical questionnaires and composite sleep disturbance data demonstrated significant differences between the lpDPN and PDPN cohorts, except the lower limb impairment, indicating that neither patient cohort had significant motor involvement (see Table 1). Following the 4-week topical 5% lidocaine treatment protocol, group composite pain scores were significantly reduced among both the lpDPN (average = 0.5; 58.3% reduction) and PDPN (average = 4.5; 30.7% reduction) cohorts (Figure 1). Clinical questionnaire data and composite sleep disturbance scores also demonstrated benefit of the 4-week topical 5% lidocaine treatment protocol among the total cohort of PDPN patients, including significant improvements on the BPI functional interference score (5.7 vs. 4.3), the NPS[10] score (56.0 vs. 43.9), and composite sleep disturbance (5.8 vs. 3.7) (see Figure 2, stars).

Individual clinical responses for each of the PDPN patients are shown (Figure 2). For the PDPN cohort, the average post-treatment composite clinical symptoms score (average of all questionnaire and sleep disturbance scores) was significantly reduced compared with the pre-treatment composite clinical symptoms score (12.97 vs. 15.97; *p* < 0.05 by paired Student's *t*-test) following topical treatment (see Table 1). As measured by QST, cold sensation pre-treatment thresholds were significantly higher (i.e., requiring a lower temperature) among PDPN patients compared with controls, as has been previously observed (55). Warm sensation thresholds were significantly higher among PDPN and lpDPN patients both before and after the topical treatment protocol, and heat pain thresholds were significantly higher among PDPN patients compared with controls following the topical treatment protocol, all standard indicators of limited functional innervation recovery following treatment (see Table 1).

### Innervation Analysis

**Figure 3:**
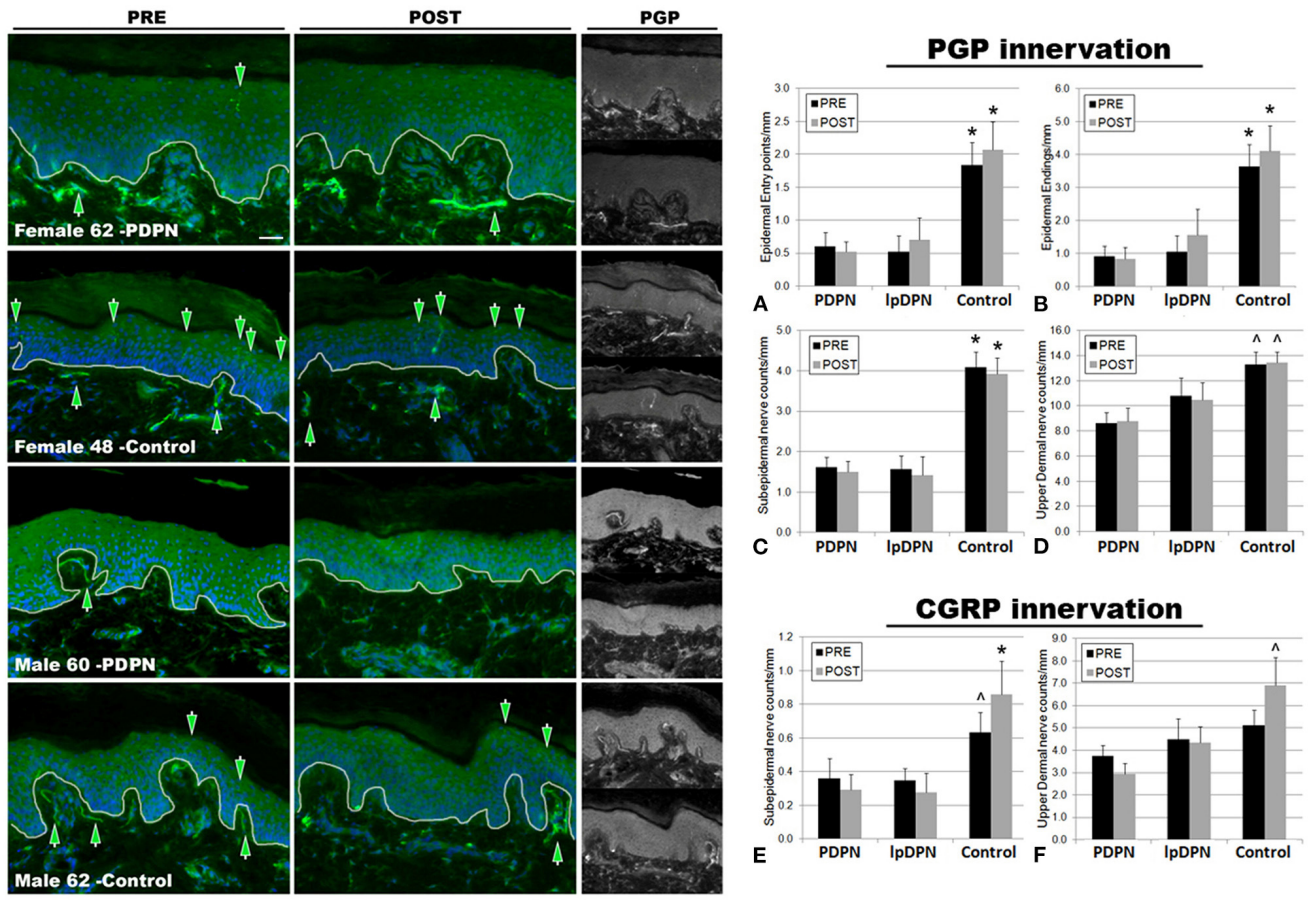
PGP9.5+ cutaneous innervation. The panels depict representative images of PGP immunolabeling and DAPI fluorescence, PRE (pre-treatment period) and POST (post-treatment period) topical 5% lidocaine patch treatment, along with the half-sized grayscale PGP images used for counting, from a female and male PDPN patient compared with control. Thin white lines in the color images indicate the dermal/epidermal junction. Mag bar = 50 μM. Analysis of intraepidermal nerve fiber (IENF) measures **(A)** epidermal entry points and **(B)** epidermal endings, as well as **(C)** subepidermal axons and **(D)** upper dermal nerves, demonstrated significant losses across all cutaneous innervation sets from both PDPN and lpDPN cohorts compared with Control subjects. No innervation measures differentiated between the PDPN and lpDPN cohorts, and the 4-week topical 5% lidocaine treatment protocol had no significant effect on the innervation loss. Analysis of CGRP-positive (peptidergic) innervation revealed similar losses among the **(E)** subepidermal and **(F)** upper dermal compartments. CGRP-positive IENF were not identified among our human cohort biopsies (see results). *significant vs. PDPN and lpDPN; ^∧^significant vs. PDPN.

To evaluate the impact of topical 5% lidocaine on cutaneous innervation, skin biopsies were immunolabeled using anti-PGP, a neuron-enriched marker well-characterized to label all cutaneous innervation (Figure 3). Detailed analysis of the small-caliber innervation entry points at the basement membrane (entry), intraepidermal nerve fiber endings (IENF), profiles of small fibers and nerves immediately subjacent to the epidermis (subepidermis), and the upper dermal axons (upper dermal) were performed using computer-assisted microscopy. The results demonstrate that PDPN is associated with a significant loss of epidermal entry points and IENF (Figures 3A,B) compared with controls, as has been described in the literature previously (6, 92). Furthermore, PDPN patients also have a significant loss of subepidermal and upper dermal nerve fiber densities (Figures 3C,D). Importantly, the lpDPN cohort also had significant losses of cutaneous innervation that were comparable to that of PDPN patients across epidermal entry points, IENF, and subepidermal compartments (Figure 3). Although the innervation density in the upper dermis of lpDPN patients trended lower than that of controls, it did not test as significantly different and also did not differentiate PDPN patients (Figure 3D). Following the 4-week topical 5% lidocaine treatment protocol, similar cutaneous innervation losses remained in epidermal, subepidermal, and upper dermal compartments in both the PDPN and lpDPN cohorts compared with control, even though pain ratings were reduced. Although the timeframe between pre- and post- biopsy collection would not provide sufficient time for IENF regeneration to occur, and lidocaine does not have any known ability to stimulate cutaneous innervation growth, the cutaneous innervation status in these cohorts documents that no changes in cutaneous innervation were responsible for the clinical benefits observed following lidocaine treatment. Furthermore, there was no correlation between PGP-positive innervation density and response to topical lidocaine treatment. MANOVA analysis revealed an overall effect, Wilks Lamda = 0.326, *F*_(14, 70)_ = 3.76, *p* < 0.001, and follow up ANOVA of individual outcome variables indicated that all variables showed significant differences. To follow up, a Bonferroni adjustment was made when evaluating differences between groups on all outcome variables.

The results indicate that all biopsy PGP innervation markers (epidermal entry points, IENF, subepidermal, and upper dermal) were able to significantly differentiate between the control and PDPN groups, and three of the four PGP measures (epidermal entry points, IENF, subepidermal) were also able to significantly differentiate between the control and lpDPN groups (Figure 3). No innervation measures differentiated the lpDPN cohort from the PDPN cohort. These results indicate that loss of cutaneous innervation (i.e., SFN) does not alone explain the pain phenotype associated with DPN. Additionally, IENF expression of CGRP was not readily observed among the human biopsies. Although described in rodent and monkey, peptidergic innervation of human epidermis has been shown by us and others to be scarce or absent, while non-peptidergic, transient receptor potential vanilloid 1 (TrpV1)-positive innervation appears to be the dominant IENF type in humans (36, 82, 93–98). Analysis of the peptidergic CGRP-positive innervation among the pretreatment biopsies revealed significant losses of subepidermal nerves across the PDPN cohort compared with control, although they did not test as significantly different for the lpDPN cohort (Figure 3E). No differences were detected among the pretreatment CGRP-positive upper dermal innervation (Figure 3F).

Following topical treatment with lidocaine, a non-specific Nav channel blocker, subepidermal CGRP-positive innervation labeling increased among controls, while slightly decreasing among the PDPN and lpDPN patients, resulting in statistically significant reductions among both cohorts (Figure 3E). Interestingly, following topical treatment with lidocaine, upper dermal CGRP-positive innervation immunolabeling was increased among controls, leading to a statistically significant difference from the PDPN patients, but not of the lpDPN cohort (Figure 3F). It is important to note that topical lidocaine, acting *via* Nav channels, likely has effects on the CGRP-positive peptidergic axons within the dermis that mediate vasogenic inflammatory responses, co-express TrpV1, and likely play a role in pain perceptions (7, 88, 99, 100).

### Keratinocyte Analysis

**Figure 4:**
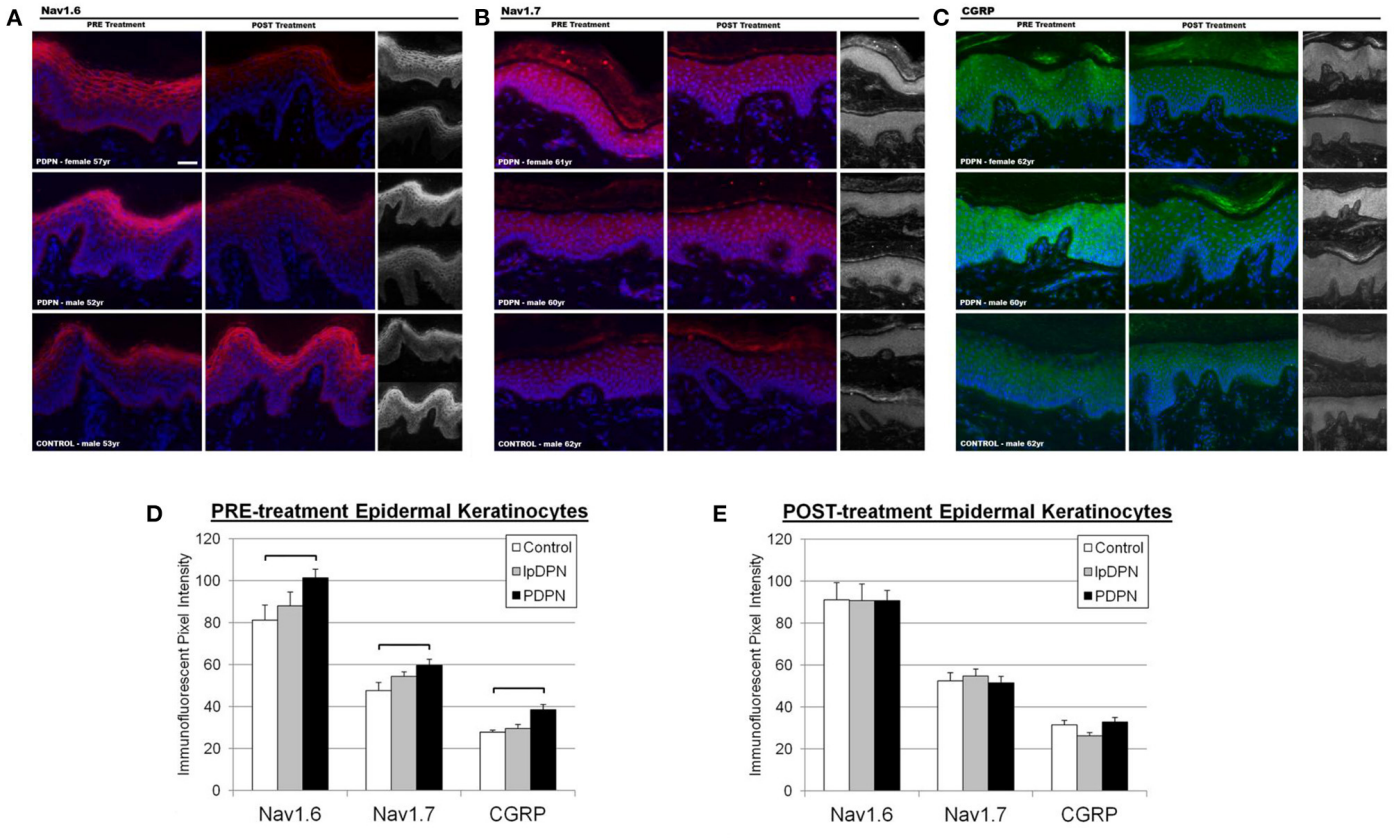
Keratinocyte biomarker immunolabeling. Epidermal keratinocyte biomarkers are significantly increased among PDPN patients compared with control biopsies. The panels depict representative images of immunolabeling for **(A)** Nav1.6, **(B)** Nav1.7, and **(C)** CGRP along with DAPI fluorescence from PRE and POST treatment biopsies, along with half-sized grayscale images used for pixel intensity measures, from age-matched female and male PDPN patients compared to a male control. **(D)** There was a significant increase in PRE-treatment Nav1.6, Nav1.7, and CGRP immunolabeling among PDPN patients compared with control (brackets indicate significant differences). **(E)** Topical lidocaine treatment acted to decrease keratinocyte biomarker levels of Nav1.6, Nav1.7, and CGRP among PDPN patients such that no differences were found among the groups POST-treatment.

To investigate keratinocyte immunofluorescent labeling pixel intensity (PI) levels for the presumptive algesic neurosignaling molecules Nav1.6, Nav1.7, and CGRP, PI from identically captured images were analyzed. Representative images of Nav1.6, Nav1.7, and CGRP immunolabeling used for analysis are shown in Figure 4. Each individual biomarker was able to differentiate PDPN patients from Control subjects (see Table 1). To evaluate the extent to which all keratinocyte biomarkers differentiate the three groups (Control, lpDPN, PDPN) prior to implementation of treatment, a MANOVA was used with follow up ANOVA of individual outcome variables. Results indicated that all variables showed significant differences. To follow up on differences between groups, a Bonferroni adjustment was made on all outcome variables. These results indicate that each keratinocyte biomarker was able to significantly differentiate between the Control and PDPN groups (Figure 4). To evaluate the post-treatment differences between groups on the biopsy markers, a MANCOVA was performed. As all pre-treatment biological marker variables showed some level of significant differences between groups, those values were used as covariates as a statistical control in evaluating post-treatment effects on the biopsy markers. Results indicated there were no significant differences between groups across the biopsy markers, Wilks' Lambda = 0.624, *F*_(14, 56)_ = 1.06, *p* = 0.41. These results indicate that while there were pre-treatment differences between groups across the keratinocyte biomarkers, after the course of treatment and adjusting for pre-existing differences on those measures, the markers no longer showed significant differences across groups after the treatment.

### Treatment Response Analysis

The lpDPN and PDPN cohorts were evaluated for response to topical lidocaine treatment based upon a 30% reduction in VAS scores. This analysis resulted in 42% (*n* = 14) of the participants being classified as non-responders (NON) and 58% (*n* = 19) as responders (RESPONDER). Subsequently, to determine what extent pre-treatment variables could predict effective response to topical lidocaine, logistic regression models were performed. The first logistic model utilized the pre-treatment keratinocyte biomarker Nav1.6 to predict responder status. The regression model was significant, χ^2^[1] = 8.67, *p* < 0.05, and *R*^2^ = 0.31. The model correctly classified 26 of 33 total participants (79%). Sensitivity was 84% and specificity was 72%. These results suggest that pre-treatment keratinocyte Nav1.6 expression was a statistically significant predictor of responder status. A second logistic model used the pre-treatment keratinocyte biomarkers Nav1.7 and CGRP as independent measures to predict responder status. The regression model was significant, χ^2^[1] = 12.50, *p* < 0.05, and *R*^2^ = 0.42. The model correctly classified 28 of 33 participants (85%). Sensitivity was 90% and specificity was 79%. These results suggest that the pre-treatment keratinocyte biomarkers Nav1.7 and CGRP were also statistically significant predictors of responder status. A third logistic model was used to evaluate the clinical measures (BPI-DLII, BPI-FPSI, NIS-LL, NPS10, NSS) as predictors of responder status. This model was not statistically significant, χ^2^[1] = 6.50, *p* = 0.27, and *R*^2^ = 0.25, indicating that these measures do not appear to have predictive value.

## Discussion

Chronic pain conditions, including PDPN, remain among the most widespread and debilitating clinical concerns, compounded by numerous treatment challenges, particularly in that existing medications offer unpredictable and/or blunted relief with a high risk of treatment-limiting side-effects (12, 79, 101). Moreover, attempts at developing safer and more effective chronic pain therapeutics have been confounded by a high failure rate in clinical trials due in part to placebo effects and the dependency on subjective patient reporting of pain perception, quality, intensity, and relief (102–104). As such, IMMPACT has identified the need for more mechanistically-relevant objective quantifiable biomarkers with which to develop more rationally-based therapeutics and to validate subjective patient pain ratings, and particularly recommending the use of skin biopsy analysis as a high priority means for identifying such biomarkers (16, 105).

This study was conducted with a biomarker discovery objective using PDPN as a chronic pain model and topical 5% lidocaine patch as an open-label proof-of-principle test. First, patient perceptions of pain severity before and after patch treatment were based on a combination of clinically used subjective measures, of which VAS and daily diary NRS are most universally accepted. The choice of biomarker assessments were based on hypotheses indicating that pathologies involving the nociceptive cutaneous innervation are the source of aberrant hyperactivity-implicated in chronic pain or based on recent discoveries that neural signaling pathologies may be occurring among epidermal keratinocytes that have been shown to regulate the sensitivity of the innervation. Recent evidence has demonstrated that keratinocytes, the major cell constituent of the epidermis and the terminal field of small-caliber intraepidermal nerve fiber (IENF) endings, have inhibitory and excitatory neurosignaling capabilities that may involve Nav subunit and/or CGRP expression (7, 36, 44, 62, 73, 74, 78, 79, 81). Keratinocytes are involved in transducing and integrating cutaneous mechanical, thermal, chemical, and nociceptive stimuli involving the release of a variety neurotransmitters, neuropeptides, neurohormones, and neuromodulators, initiated through several types of chemically- or physically-activated receptors and ion channels (7, 36, 62). Keratinocytes may directly activate or inhibit primary sensory endings within and beneath the epidermis and/or exert an excitatory or inhibitory modulation of their sensitivity (71, 76). Therefore, pathologies among keratinocyte neurosignaling properties may contribute to the increased nociceptive activity driving various chronic pain sensations (i.e., shocking, stabbing, pricking, burning).

Our assessments of IENF density of PGP labeling confirmed prior reports of a consistent and significant paradoxical reduction (i.e., SFN) among the PDPN patients as compared to that in normal subjects, but there was no significant correlation between the severity of IENF reduction and patient perceptions of pain severity (2, 6, 25, 47, 48, 51). Importantly, significant IENF reductions also occurred among the lpDPN patients that was comparable to losses in PDPN patients. Our study added new information that significant and comparable reductions also occur among the upper dermal and subepidermal innervation among both the lpDPN and PDPN patients, and furthermore that no recovery of innervation was detected after patch treatment—-even among patients who subjectively reported decreased pain perception. Unlike in other species, including Rhesus monkeys, CGRP could rarely be detected among the IENF in normal subjects. However, our assessments of CGRP fiber densities in the upper dermis revealed a significant reduction of CGRP fibers and no post-patch innervation regeneration. Although many other biomarkers have yet to be investigated, we found no evidence related to differences in PGP and CGRP labeled innervation densities, proportions of CGRP, or regeneration that significantly correlated with patient subjective pain ratings.

Also seen in other chronically painful peripheral neuropathies, electrophysiological evidence of increased sensitivity and spontaneous activity among the remaining innervation is the basis for the irritable nociceptor hypothesis of spontaneous hyperactivity. This prevailing theory for the seemingly paradoxical loss of nociceptors is based on electrophysiological evidence that chronic neuropathic pain may be mediated by a hypersensitivity of the remaining innervation. Importantly, comparable losses among both lpDPN and PDPN indicate that reduced innervation is not itself a precipitator of chronic pain among diabetic patients. There are different reasons that reductions in presumed C-fiber nociceptors occurs among a variety of chronic pain afflictions, of which some are obvious such as after nerve traumas or infections such as acute herpes zoster that may be consistent triggers for pathological chronic pain mechanisms. The loss of innervation in lpDPN patients may be due to any of the numerous diabetic pathophysiologic mechanisms, including hyperglycemia, chronic inflammation, and ROS generation that are detrimental to vulnerable C-fibers, yet without inducing pain (53, 66–70).

The basis for our choice of using the lidocaine patch as our test therapeutic is that we have previously demonstrated that some Nav subtypes are also normally present on human keratinocytes and that keratinocyte Nav activation can result in a release of ATP *in vitro*, which is a direct activator of small fiber axons (44, 81, 100, 106). Moreover, we have shown Nav channel increases and there is *de novo* labeling among epidermal keratinocytes of painful skin from human CRPS type 1 patients, as well as in herpes zoster (HZ) subjects that had resolved with severe PHN (44). In those studies, both cohorts had reduced epidermal innervation. However, keratinocyte Nav expression was not increased in the HZ patients with resolved severe PHN, indicating that keratinocyte Nav expression may be a predictable marker of PHN chronic pain, unlike innervation density. Therefore, the analgesic effect of lidocaine may involve blocking keratinocyte Nav channel activity.

Here, we assessed the relative levels of cutaneous innervation density and keratinocyte immunolabeling for Nav1.6, Nav1.7, and CGRP in skin biopsies from PDPN, lpDPN, and control subjects, and sought to determine if a 4-week topical 5% lidocaine treatment protocol would provide relief of PDPN, coincident with alterations of these skin biopsy biomarkers. In pretreatment biopsies, epidermal keratinocyte immunoreactivity for Nav1.6, Nav1.7, and CGRP were all significantly elevated among the PDPN cohort compared with control. Importantly, the increased levels of keratinocyte Nav1.6, Nav1.7, and CGRP immunoreactivity in PDPN patients were all significantly reduced to approximate control levels following topical 5% lidocaine treatment, whereas treatment made no significant improvements in innervation. Additionally, response analysis revealed that the elevated PRE-treatment keratinocyte Nav1.6, Nav1.7, and CGRP immunoreactivity positively correlated with the responder population, indicating that these keratinocyte biomarkers may likely be directly involved in the pain pathophysiology. Indeed, we have also previously shown a role for CGRP in keratinocyte algesic mechanisms, and recently several classes of CGRP-inhibitors have been approved for migraine, and which may have benefit to other pain disorders, including PDPN, fibromyalgia, ME/CFS, PTSD, and others with SFN, including PHN and potentially similar post-viral lesions from long-Covid-19 (7, 36, 60, 65, 107–109).

This current study was an open-label proof of principle study that was not designed to assess efficacy of the lidocaine patch in PDPN, *per se*, and did not include a non-drug treated group. Rather, this was designed as an investigative clinical study to test for cutaneous differences in pain and non-pain diabetic neuropathy patients compared with control subjects, and particularly to determine if there were any correlations of keratinocyte biomarkers with pain and/or response to treatment. Like all clinical research studies, there are certain limitations to the strength of the data generated here. First, the data are based on a small cohort size, as a pilot investigation. The study was performed at a single site, and the patient testing and clinical workups performed by the same physicians, which indirectly also adds a certain degree of strength to the results. However, these results should be confirmed in a large cohort study. Secondly, the tissue analysis and IENF determinations were not performed in the more standard distal leg location, but instead were taken from the dorsolateral foot. Although this makes these IENF measures not directly comparable with common reference data published in the literature, the increased wealth of morphologic data associated with biopsy analysis from glabrous skin has been well-established in previous publications. Indeed, a strength of the data resides in the novel comprehensive biomarker analysis of complex glabrous skin tissue. As well, another potential limitation of any clinical pain study is a placebo effect. Placebo effects are often cited as reason why clinical pain drugs fail in the clinical trial—a group who did not get drug (placebo group) shows a response that mimics the drug treated group, thereby eliminating any statistical differences. This study utilized an open-label design and all patients and control subjects received drug treatment. There was not a true placebo group for this study. Therefore, the positive (analgesic) clinical responses of the responder subgroup of the PDPN cohort are presumed to be pharmacologic in nature (i.e., lidocaine-mediated), although the chance for a placebo effect related to subjective pain symptoms, in the absence of pharmacology, remains a possibility.

Altogether, the results indicate that cutaneous innervation loss (clinically diagnosed as SFN) does not fully explain the pain phenotype in diabetic neuropathy. That efficacious topical treatment associated with keratinocyte Nav1.6 and Nav1.7 channels and CGRP immunolabeling, and specifically that epidermal keratinocyte biomarker levels correlated with pain reduction, implicates these mechanisms as potential contributors to the pain phenotype. Continued clinical research with comprehensive skin biopsy analysis will continue to uncover novel biomarkers and drug targets for chronic neuropathic pain, including keratinocyte/neuron interactions, which may be treated through rationally designed topical therapies.

## Data Availability Statement

The original contributions presented in the study are included in the article/supplementary materials, further inquiries can be directed to the corresponding author/s.

## Ethics Statement

The studies involving human participants were reviewed and approved by Albany Medical College. The patients/participants provided their written informed consent to participate in this study.

## Author Contributions

PA, JW, CA, and FR were involved in all aspects of the study. GH was involved in morphologic data collection, analysis, and interpretation. GH, ER, MD, MC, and JB were involved in clinical and morphologic data collection, analysis, and writing. All authors contributed to the article and approved the submitted version.

## Funding

Funding for this clinical pain research project was provided, in part, through an investigator-initiated grant mechanism from Endo Pharmaceuticals (awarded to CA), by the Center for Neuropharmacology and Neuroscience at Albany Medical College, and by Integrated Tissue Dynamics, LLC (INTiDYN). All authors conceived, planned, performed, analyzed/interpreted the data, and documented the findings without input from Endo Pharmaceuticals.

## Conflict of Interest

GH, ER, and MD are employed by INTiDYN. JB was a paid consultant of INTiDYN. PA and FR are owners of INTiDYN. INTiDYN remains a private company with no stock or options conflicts. The remaining authors declare that the research was conducted in the absence of any commercial or financial relationships that could be construed as a potential conflict of interest.

## Publisher's Note

All claims expressed in this article are solely those of the authors and do not necessarily represent those of their affiliated organizations, or those of the publisher, the editors and the reviewers. Any product that may be evaluated in this article, or claim that may be made by its manufacturer, is not guaranteed or endorsed by the publisher.
